# Gaining insight into benzodiazepine prescribing in General Practice in France: a data-based study

**DOI:** 10.1186/1471-2296-12-28

**Published:** 2011-05-11

**Authors:** Sophia Rosman, Le Vaillant Marc, Pelletier-Fleury Nathalie

**Affiliations:** 1 INSERM U988, CNRS, UMR 8211, 7 rue Guy Moquet, 94801 Villejuif Cedex, France; 2 EHESS, 54 bd Raspail, 75006 Paris, France; 3 Université Paris-Descartes, 45 rue des Saints-Pères, 75270 Paris cedex 06, France

## Abstract

**Background:**

In recent decades, benzodiazepine (BZD) prescriptions have been called into question in most European countries by physicians and health authorities alike, and guidelines on medical indications and treatment duration have been established to avoid long-term use and dependency. In France, many public policy measures have been implemented as BZDs are among the most prescribed medications. General practitioners (GPs) were identified by the Caisse d'Assurance Maladie (the French public health insurance fund) as high prescribers for these drugs. In this context, the aim of the study was to determine GPs' rates and to identify correlates of BZD and Z-drugs prescribing.

**Methods:**

Data on patient characteristics, diagnoses and BZD prescriptions were drawn from French GPs' electronic medical records. These were accessed via the database which the *Société Française de Médecine Générale*, the French Society of General Practice, has been compiling since 1993 in a network of 90 GPs working mainly in solo practices. The participants in this network routinely register data in their daily practice. The present study examined 51,216 patients from 52 GP practices and we performed a multivariate logistic regression. The dependent variable was whether a patient was prescribed BZD at least once during 2006.

**Results:**

In the present study, 12.5% of patients older than 18 were prescribed BZDs at least once during 2006 and the average (SD) was 2.6 (2.4) BZD prescriptions/patient/year. The adjusted odds (confidence interval) of having at least one BZD prescription were 1.20 (1.10 - 1.30) in patients older than 65; 1.05 (1.01 - 1.10) in women; 1.25 (1.17 - 1.33) in patients with associated comorbidities (cardiovascular diseases) and 1.76 (1.62 - 1.92) in heavy consumers of health care (more than 4 consultations with a GP per year).

**Conclusions:**

The present study showed the persistence of high rates of BZD prescription by GPs, particularly in women and older patients, which highlights the difficulties of implementing effective public policies and the necessity of using new approaches enabling doctors and patients to understand the true relative advantages, disadvantages, and consequences of using these drugs and of non-pharmaceutical treatments.

## Background

In recent decades, BZD prescriptions have been called into question in most European countries by physicians and health authorities alike, and guidelines on medical indications and treatment duration have been established to avoid long-term use and dependency [1,2]. In France, many public policy measures have been implemented as BZDs are among the most prescribed medications [3]. The *Agence Française de Sécurité Sanitaire et des Produits de Santé *(AFSSAPS), an institution in charge of the risk-benefit assessment of the use of healthcare products, initiated various actions to promote the safe use of BZDs. These actions, which were supported by working groups and ad hoc surveys, led to the establishment of good practice recommendations [4].

Yet, in a recent report on the proper use of psychotropic medications, it was shown that these drugs were often inappropriately prescribed (in terms of users, indications, duration of treatment) [5]. General practitioners (GPs) were identified by the Caisse d'Assurance Maladie (the French public health insurance fund) as high prescribers for these drugs [6]. Gaining insight into GPs' BZD prescribing seemed appropriate in this context.

The aim of this study was to determine GPs' rates and to identify correlates of BZD and Z-drugs prescribing in a large database in general practice in France.

## Methods

### General practitioner sampling

Data on patients, diseases and related health problems and BZD prescriptions were drawn from French GPs' electronic medical records. These were accessed via the database which the *Société Française de Médecine Générale*, the French Society of General Practice, has been compiling since 1993 in a network of 90 GPs working mainly in solo practices (SFMG-DB). The participants in this network routinely register data in their daily practice. They are largely representative of the French GP population [7], although a comparison with data from the Ministry of Health shows that doctors working in rural areas were under-represented [8]. We studied the practices of the 52 GPs for whom complete information with regard to prescriptions was available during 2006 (Commission Nationale de l'Informatique et des Libertés (CNIL) - approval n° 311668).

### Patient registration

The 52 GPs had cared for a total of 75,367 patients whose age and sex distribution did not differ significantly from that of the population as a whole. For the present data-based study, we only selected patients older than 18, which gave us a sample of 51,216 patients.

### Codes for diseases and related health problems

In the SFMG-DB, diseases and related health problems are coded using the Dictionary of Consultation Results (DCR), which has been validated in France [9]. The corresponding codes in the International Classification of Diseases, 10th Revision, Clinical Modifications (ICD-10-CM) were also mentioned. The diseases and related health problems selected as indications for BZD are mentioned in Table 1. We also collected data on other diseases and related health problems that are frequently encountered in general practice (in the top 10 with insomnia/anxiety/depression) [7] (also see Table 1).

**Table 1:** Corresponding codes in the Dictionary of Consultation Results and the International Classification of Diseases, 10th Revision, Clinical Modifications

disease	Dictionary of consultation result (DCR)	International classification of diseases (ICD-10-CM)
Insomnia	DCR 742	ICD F51.0
Stress reaction	DCR 752	ICD F43.9
Mild depressive	DCR 739	ICD F32.0
Depression	DCR 727	ICD F32.9
Anxiety	DCR 859	ICD F41.9
Hypertension	DCR 826	ICD I10
Hyperlipidemia	DCR 740	ICD E78.5
Type 2 diabetes	DCR 818	ICD E11
Lumbar region pain	DCR 850	ICD M54.5
Arthrosis	DCR 715	ICD M19.9
Arthropaties/Periarthropathies	DCR 828	ICD M25.9

### BZD prescriptions

In the SFMG-DB, prescriptions are coded according to the Anatomical Therapeutic Chemical (ATC) Classification System (WHO, 2006). We retained four therapeutic categories: (i) BZD-derivative anxiolytics: ATC group N05BA (e.g., diazepam, oxazepam, lorazepam); (ii) BZD-derivative hypnotics and sedatives: ATC group N05CD (e.g., flurazepam, nitrazepam, temazepam); (iii) BZD-derivative anti-epileptics: ATC group N03AE (e.g., clonazepam); and (iv) Z-drugs: ATC group N05CF (e.g., zopiclone and zolpidem).

### Data analysis

The chi-square test and Student's *t *test were used to compare cross-classified and continuous variables, respectively. We performed a multivariate logistic regression, and the following covariates were introduced into the model: age (<65/≥65), gender (M/F), level of healthcare consumption (<4/≥4 consultations/year) and presence of comorbidities [at least one consultation for anxiety and/or depression and/or insomnia during the year (Y/N), at least one consultation for hypertension and/or hyperlipidemia and/or type 2 diabetes during the year (Y/N) and at least one consultation for lumbar region pain and/or arthrosis and/or arthropathies during the year (Y/N)]. The interactions between all these covariates were tested and included in the model when statistically significant. The dependent variable was whether a patient was prescribed BZD at least once during 2006. The data had a two-level hierarchical structure, with individuals nested in GPs. Within GP clustering was taken into account by estimating the fixed coefficients and their standard errors using the method of generalised estimated equation (GEE) [10]. SAS software was used for the analyses [11].

## Results

### Patient characteristics

**Figure 1:**
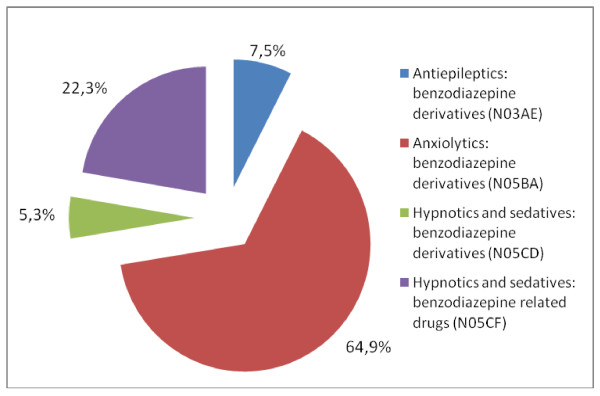
**Distribution of BZDs prescriptions in the study population, 16886 in all, according to the therapeutic categories**.

Patient characteristics in the study population (n = 51,216) are summarised in Table 2. In our patient sample, 12.5% (n = 6,405) were prescribed BZDs at least once during 2006, and the average (SD) number of consultations per patient with at least one BZD prescription was 2.6 (2.4). Figure 1 presents the distribution of BZD prescriptions - 16,886 in all - according to the therapeutic categories. The most frequently prescribed medications by French GPs were from the ATC groups N05BA (e.g., diazepam, oxazepam, lorazepam), which accounted for 64.9% of all BZD prescriptions, and N05CF (e.g., zopiclone and zolpidem), which accounted for 22.3%. Medication from the ATC group N05CF was over-represented in patients 65 and older (24.4% vs. 21.1% in patients under 65).

**Table 2:** Patients' characteristics in the study population (n = 51,216)

Patients 18+	
Mean age in years (SD)	45,7 (18,4)
% of patients of 65 or older	17.5
% females	54,5
Nb consultations/patient/year (SD)	3,2 (2.7)
% patients with > 4 consultations/year	24
Nb consultations/patient/year with at least one prescription (SD)	2,6 (2.3)
% patients with at least one prescription	91,3
% of patients with at least one BZD prescription	12,5
% patients	
- with anxiety	3.7
- with depression	10.3
- with insomnia	4.1
- with cardiovascular diseases	18.2
- with arthropathic diseases	22.4

### Univariate analyses

Table 3 presents a comparison of the descriptive characteristics of patients in the study population according to whether or not they were prescribed BZDs. Anxiety, insomnia and depression were coded in respectively 16.9, 20.9 and 41.3% of the patients who were prescribed BZDs. Patients of 65 and older were over-represented in this latter group compared with those who were not prescribed BZDs (27.1% vs. 16%, P < 0.001). Table 4 compares the descriptive characteristics of patients who were prescribed BZDs according to age (under or over 65). Among patients 65 and older, 19.5% were prescribed BZDs at least once.

**Table 3:** Comparison of the descriptive characteristics of patients in the study population (n = 51,216) according to whether or not they were prescribed BZDs

Patients 18+	Without BZDs prescriptions (n = 44,811)	With BZDs prescriptions (n = 6,405)	P value
Mean age in years (SD)	44.5 (18.2)	53.5 (17.1)	P < 0.001
% patients of 65 or older	16.0	27.1	P < 0.001
% females	53.1	64.1	P < 0.001
Nb consultations/patient/year (SD)	2.9 (2.4)	5.3 (3.3)	P < 0.001
% patients with > 4 consultations/year	19.8	52.6	P < 0.001
% patients			
- with anxiety	1.9	16.9	P < 0.001
- with depression	5.8	41.3	P < 0.001
- with insomnia	1.7	20.9	P < 0.001
- with cardiovascular diseases	20.5	36.4	P < 0.001
- with arthropathic diseases	16.8	27.7	P < 0.001

**Table 4:** Comparison of the descriptive characteristics of patients who were prescribed BZDs (n = 6,405) according to age (less than 65/65 or more)

Patients 18+	< 65 (n = 4,667)	≥ 65 (n = 1,738)	P value
Mean age (SD)	45.2 (11.5)	75.5 (6.9)	P < 0.001
% female	62.7	67.8	P < 0.001
Nb consultations/patient/year (SD)	5.15 (3.4)	5.98 (3.1)	P < 0.001
Nb consultations/patient/year with at least one prescription (SD)	4.4 (2.9)	5.0 (2.7)	P < 0.001
% patients with > 4 consultations/year	50.0	63.9	P < 0.001
% patients			
- with anxiety	17.8	14.5	p = 0.002
- with depression	46.8	26.7	P < 0.001
- with insomnia	17.8	29.2	P < 0.001
- with cardiovascular diseases	24,9	67.3	P < 0.001
- with arthropathic diseases	23.2	39.8	P < 0.001
Mean nb of BZDs prescriptions/year (SD)	2.4 (2.3)	3.3 (2.3)	P < 0.001
			
% patients with at least one prescription of			
- N05BA	75.6	66.9	P < 0.001
- N05CD	5.3	6.3	NS
- N03AE	8.1	10.9	P < 0.001
- N05CF	25.9	27.6	NS

### Logistic regression model

The detailed results of the logistic regression model are summarised in Table 5. The adjusted odds (confidence interval) of having at least one BZD prescription were 1.05 (1.01 - 1.10) in women, 1.25 (1.17 - 1.33) in patients with associated co-morbidities (e.g., cardiovascular diseases), and 1.76 (1.62 - 1.92) in heavy consumers of healthcare (more than four consultations with a GP per year). Even after adjustment for the different covariates introduced into the model, the effect of age on BZD prescriptions was still significant (adjusted OR = 1.20 (1.10 - 1.30)).

**Table 5:** Results of the logistic regression model for the dependent variable: whether or not a patient was prescribed BZDs, at least once, during the year 2006

	Coefficient	P value	Odds ratio	95% CI	
Intercept	-1.71	<0.0001			
Gender (ref = male)	0.05	0.009	1.05	1.01	1.10
Age (ref = <65)	0.18	<0.0001	1.2	1.11	1.3
Health care consumption (ref = >4 consultations/year)	0.57	<0.0001	1.76	1.62	1.92
Cardiovascular diseases (ref = no)	0.22	<0.0001	1.25	1.17	1.33
Arthropathic diseases (ref = no)	0.07	0.009	1.07	1.01	1.13
Anxiety (ref = no)	1.98	<0.0001	7.29	5.86	9.05
Depression (ref = no)	1.95	<0.0001	7.03	6.03	8.2
Insomnia (ref = no)	2.22	<0.0001	9.25	7.26	11.7
Gender*age	0.24	<0.0001			
Gender * Health care consumption	- 0.09	0.017			
Age* Cardiovascular diseases	-0.14	0.009			
Age* Depression	-0.51	<0.0001			
Anxiety *Depression	-1.19	<0.0001			
Depression*Insomnia	- 0.68	0.007			

## Discussion

Despite the efforts made by healthcare authorities, physicians' unions and healthcare professionals to curb BZD use, our study still shows a rate of BZD prescription of around 12%, which is close to what it was several decades ago [6].

Age was a main determinant of BZD prescribing: elderly patients received more BZD prescriptions than younger patients. This finding was consistent with data from other studies [12-15]. It is not explained, as suggested by Morin et al [16], by more frequent visits with GPs since the association was still significant after controlling in our model for the effect of the number of consultations with a GP per year. Other reasons could explain BZD prescribing in elderly people, in particular the perception of elderly people with regard to the process of ageing and the use of BZDs in this process. Collin showed that older people considered themselves to be undergoing a process of deterioration and that only pharmaceuticals, in particular BZDs, could treat the related stress and anxiety [17]. Moreover, physicians also view the process of ageing as one of loss, marked by physical and mental decline as well as by social and emotional isolation [18]. From this perspective, BZDs are seen by doctors as "compassion pharmaceuticals" that serve to relieve the suffering of elderly patients. This contrasts with the general message put out by the experts, which focuses on the necessity of reducing BZD prescriptions for elderly people because of their considerable side effects [16].

Being a female was also significantly predictive in our model. This result was consistent with prior reports [19,20]. It has been shown that most female users took BZDs to treat anxiety (ranging from fears of dying to claustrophobia), to be "calm" and "relaxed" in daily situations [21]. According to Van der Waals et al, women also received more often than men BZDs for not legitimate indications (like headache and general fatigue) [19]. In a recent study carried out in general population, it was confirmed that potentially inappropriate drug use (all types of drugs confounded) were more common in women (24,6%) than in men (19,3%). In particular, female sex was highly associated with inappropriate psychotropic use (eg, long-acting BZDs) [20]. BZD withdrawal can be a traumatic process for both patients and doctors. This reputation is largely undeserved if the process is carried out judiciously [22]. Although different approaches have been assessed, BZD withdrawal remains a key issue that is still difficult to resolve in routine care [23].

Suffering from somatic chronic disease was also associated with BZD prescribing [24]. This could be explained by the fact that somatic chronic disease causes anxiety and insomnia [25,26] which are associated with BZD prescribing [24]. However, the effect that we detected for somatic diseases was independent of anxiety and insomnia diagnoses. This finding was also underlined by Mant et al. but they did not put forward a clear explanation [12]. Our hypothesis was that diagnoses of anxiety and/or insomnia were not always coded in the database when a BZD was prescribed to patients with a somatic disease.

Finally, our findings showed that being a heavy consumer of health care was also associated with BZD prescriptions. But the lack of information on the use of specialist care, in the French health care context in 2006, prevented us from correctly interpreting this finding. Indeed, patients could consult a specialist (e.g., for cardiovascular diseases or arthropathies) without necessarily going through a GP. This causes a substitution effect between the two types of doctors and reduces the use of GPs [27]. In our model, we assumed that if we had adjusted for the number of visits to a specialist during the year, the independent effect of heavy healthcare consumption on BZD prescribing would have disappeared.

### Limitations of the study

Firstly, the SFMG-DB does not include information on the duration of prescribed BZD treatment. Therefore, we could not distinguish between short-term and continuous prescriptions. Although this could limit the conclusions of our study, our model was capable of identifying correlates of BZD prescriptions in patients consulting GPs. Secondly, data on doctors' characteristics were not available in the SFMG-DB at the time of our study. This precluded a study of interactions between patients and doctors in a multilevel approach. Nevertheless, within GP clustering was taken into account in the statistical analysis by estimating the fixed coefficients and their standard errors using the GEE method. Thirdly, because in 2006 it was not yet mandatory for patients to be registered with their GP, we only had access to information on consulting patients. Therefore, we should be cautious in comparing the rates of BZD prescribing of the present study with those of previously published studies conducted in populations of patients registered with their GPs.

## Conclusions

The findings of our study are consistent with studies on BZD prescribing in other European countries. BZD prescribing is significantly related to age, gender, chronic illness and health care consumption. Despite public policy actions, and good practice recommendations, the BZD prescribing rate remains the same as several decades ago. Until now, most of the actions organised by Health Authorities to decrease BZD use and prescriptions applied to GPs and their prescribing behaviour. As suggested by Siriwardena et al, new approaches could be developed enabling doctors and patients to understand the true relative advantages, disadvantages, and consequences of using these drugs on the one hand and of non-pharmaceutical treatments on the other hand [28]. Finally, an inter-professional dialogue between GPs, psychiatrists, and pharmacists could also provide useful resources about the use and/or the withdrawal of BZD by patients [29].

## Competing interests

The authors declare that they have no competing interests.

## Authors' contributions

SR participated in the design of the study, contributed to discussion, wrote manuscript and reviewed/edited manuscript. MLV participated in the design of the study, performed the statistical analysis, contributed to discussion and reviewed/edited manuscript. NPF contributed to discussion, wrote manuscript and reviewed/edited manuscript. All authors read and approved the final manuscript.

## Pre-publication history

The pre-publication history for this paper can be accessed here:

http://www.biomedcentral.com/1471-2296/12/28/prepub
